# Improving galegine production in transformed hairy roots of *Galega officinalis* L. via elicitation

**DOI:** 10.1186/s13568-022-01409-7

**Published:** 2022-06-03

**Authors:** Maryam Khezri, Rasool Asghari Zakaria, Nasser Zare, Mohammad Johari-Ahar

**Affiliations:** 1 Department of Crop Production and Genetics, Faculty of Agriculture and Natural Resources, University of Mohaghegh Ardabili, Ardabil, Iran; 2 Department of Medicinal Chemistry, School of Pharmacy, Ardabil University of Medical Sciences, Ardabil, Iran

**Keywords:** Galegine, HPLC, *Rhizobium rhizogenes*, Secondary metabolite

## Abstract

*Galega officinalis* L. is an herbaceous legume used to treat symptoms associated with hyperglycemia or diabetes mellitus because of its dominant alkaloid, galegine. In this study, we induced hairy roots in this plant using *Rhizobium rhizogenes* strain A4, and investigated the effect of type, concentration, and duration of elicitor application on galegine content and some phytochemical characteristics in the hairy roots. Hence, the best growing hairy root line in terms of growth rate was selected and subcultured for treatment with elicitors. Then, at the end of the log phase of growth, chitosan (100, 200, and 400 mg/L), salicylic acid (100, 200, and 300 mM), and ultrasound (1, 2, and 4 min) were applied to hairy roots culture medium. High-performance liquid chromatography (HPLC) showed that the content of galegine was significantly increased after elicitation compared with the control. Thus, the highest content of galegine (14.55 mg/g FW) was obtained 2 days after elicitation when ultrasonic waves were applied to the hairy root culture medium for 4 min. Also, elicitation resulted in a significant increase in the content of total phenol, flavonoid, H_2_O_2_ and MDA compared with the control. So that the highest total flavonoid content was obtained in hairy roots that were treated with ultrasonic waves for 4 min and harvested 2 days after elicitation; while, application of 400 mg/L chitosan for 4 days resulted in the highest total phenol (16.84 mg/g FW).

**Graphical Abstract:**

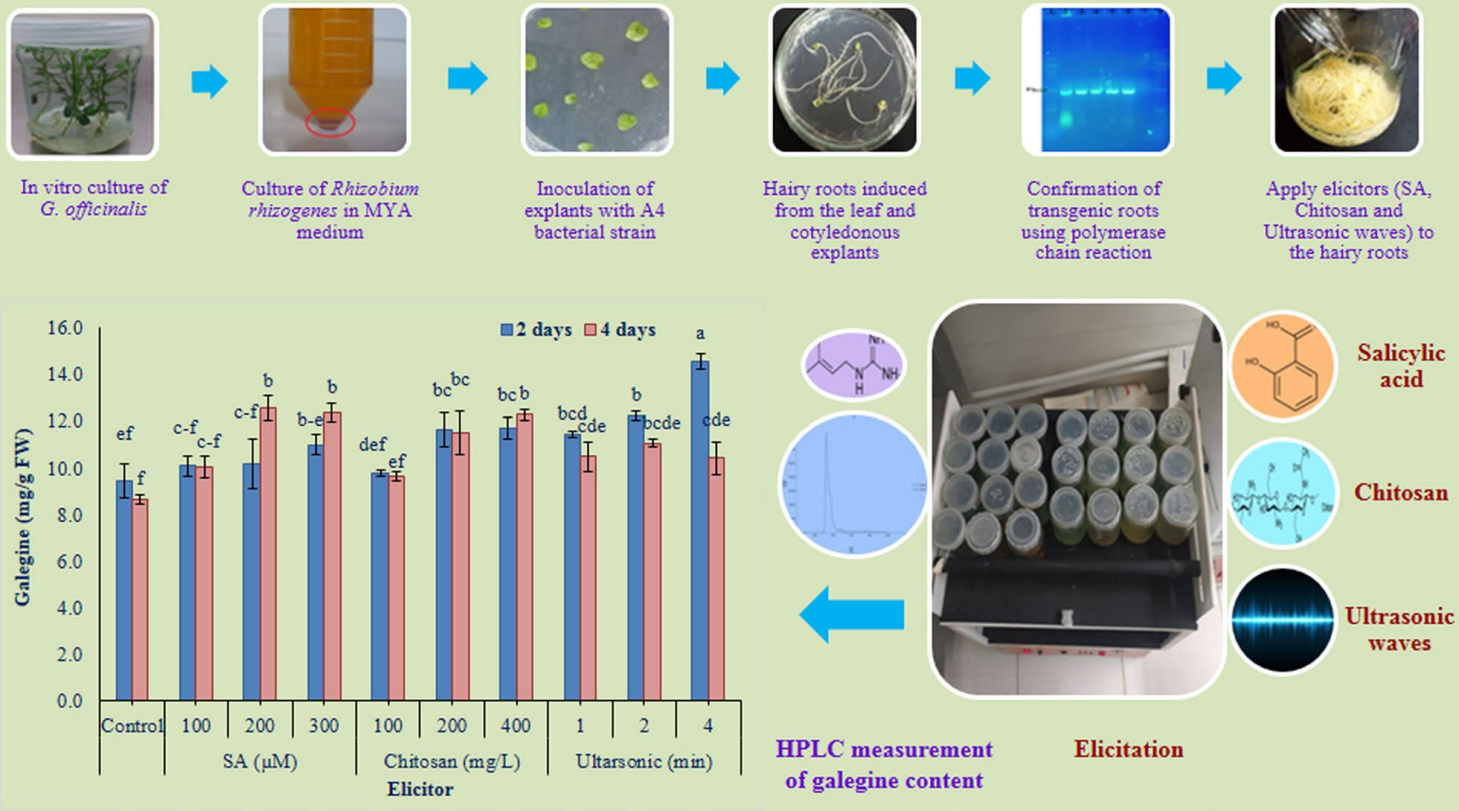

## Key points

- This is the first report on hairy root induction and elicitation in *G. officinalis*.
- Hairy root cultures can be a potential source for galegine production.
- Ultrasonic wave was more effective in increasing galegine content.

## Introduction

*Galega officinalis* L. is an herbaceous legume (Luka and Omoniva 2012) that contains medicarpin, sativan (Le Bail et al. 2000), flavonol triglycosides, kaempferol, quercetin (Champavier et al. 1999; Peiretti and Gai 2006), fatty acids (Peiretti and Gai 2006), glycosides, phenols, resins, terpenes, steroids (Okhale et al. 2010), and alkaloids (Yang 2011). Containing these compounds, *G. officinalis* has various medicinal properties such as diuretic, antibacterial, and antidiabetic (Karakaş et al. 2012), anti-inflammatory (Chevallier 1998), weight-reducing (Mooney et al. 2008), anticancer, mutation-inhibiting, antiviral, and lactate-forming effects (Le Bail et al. 2000; González-Andrés et al. 2004). The plant has been traditionally used primarily to treat symptoms now associated with type 2 diabetes (Bailey and Day 2004). The antidiabetic potential of this plant is related to a guanidine alkaloid known as galegine. Indeed metformin, a well-known, safe, and the inexpensive drug was produced from galegine of *G. officinalis* in 1950 (Chan et al. 2010; Yang 2011).

Since secondary metabolites are useful to human health as a major component of food and drugs, plants are important sources for the discovery of new medicine (Amani et al. 2021; Delgoda and Murray 2017). The extraction of these useful biochemicals through conventional farming methods and the large-scale vulnerability of the plants has resulted in falling them into the category of endangered plants (Gantait and Mukherjee 2020). To alleviate this problem, in vitro culture technology are particularly useful for high-value products that are threatened by overexploitation (Krol et al. 2021). In vitro plant cell or tissue cultures can be used throughout the whole of the year to produce high and stable yields of desired chemicals under controlled and sterile conditions (Krol et al. 2021). Hairy roots are caused by the infestation of a soil-born bacterium called *Rhizobium rhizogenes* (formerly called *Agrobacterium rhizogenes*) in a variety of plants (Gutierrez-Valdes et al. 2020). This technology has been used in recent years for the genetic enhancement of medicinal and aromatic plants and also for obtaining economic products in the form of secondary metabolites, which are of great importance due to their ethnobotanical and pharmaceutical properties (Gantait and Mukherjee 2020). Due to genetic and biosynthetic stability (Häkkinen et al. 2016; Peebles et al. 2009), the high growth rate in growth regulator-free media, and production consistency in response to elicitors, hairy root culture is more suitable than cell suspension culture for the production of specialized metabolites (Halder et al. 2019). Based on their accumulation pattern, secondary metabolites can be classified into constitutive, preformed, and inducible metabolites (Hartmann 2007). Induced metabolites (such as terpenes, phytoalexins, and alkaloids) are metabolites whose biosynthesis is activated or increased in response to internal or external factors (Halder and Jha 2020; Matsuura et al. 2018).

Elicitation is one of the most effective and widely used biotechnological tools for the induction of secondary metabolites in plant tissue cultures (Akula and Ravishankar 2011; Wang and Wu 2013). The term elicitor originally refers to molecules capable of stimulating phytoalexins but is now commonly used to refer to molecules that arouse any type of plant defense response (Nürnberger 1999) and lead to increased synthesis and accumulation of secondary metabolites (Halder et al. 2019). Numerous factors influence the change in secondary metabolite content during elicitation, including type and concentration of elicitor, duration of exposure, type of culture, cell line, the composition of the medium, age or stage of the culture at the treatment time, and the presence or absence of growth regulators (Dhiman et al. 2018; Halder et al. 2019; Kaur and Pati 2018; Naik and Al-Khayri 2016).

Salicylic acid (SA) is a natural phytohormone produced by plants during their normal metabolic processes (Narayani and Srivastava 2017) and in response to stress (Szymczyk et al. 2021) and is involved in the signal transduction cascades of plant defense responses (Giri and Zaheer 2016; Ramirez-Estrada et al. 2016). Considering the biosafety of the products, the use of these nontoxic molecules as elicitors to improve the yield of phytochemicals in vitro plant cultures has been highly recommended (Gai et al. 2019).

Chitosan, a natural biopolymer found in shrimp or insect shells (Malerba and Cerana 2015) is a low-cost, safe, and nontoxic compound (Chayjarung et al. 2021) that has the potential to induce the production of secondary metabolites in plants (Jiao et al. 2018). Chitosan is known to potentially trigger plant defense responses, forming a semipermeable film around plant tissue (Ghauth et al. 1994; Pérez-Alonso et al. 2012). It also induces the synthesis of pathogenesis-related (PR) proteins and several defense enzymes such as phenylalanine ammonia lyase and peroxidase (Ferri and Tassoni 2011).

Ultrasonic waves, often considered “mechanical waves” have been used to enhance the production of valuable secondary metabolites and stimulate the release of these compounds into the surrounding medium in plant cell cultures (Alsoufi et al. 2019a; Wang et al. 2006). High energy ultrasound waves are usually destructive to biological material, but low energy ultrasound waves have been used to stimulate plant defense responses and secondary metabolite production (Wu and Lin 2002; Zare et al. 2014). Depending on the plant species, ultrasonic waves can significantly increase the synthesis of secondary metabolites (Dörnenburg and Knorr 1995; Taherkhani et al. 2019). The advantages of ultrasound are that it is extremely inexpensive and technically feasible, without the need to add external chemical substances, and with a lower risk of microbial contamination (Alsoufi et al. 2019a).

In recent years, numerous elicitors have been shown to enhance the accumulation of various secondary metabolites in many plant species. For example, salicylic acid increases papaverine and noscapine in *Papaver armeniacum* L. (Naeini et al. 2021), and tanshinone in *Salvia przewalskii* Maxim (Li et al. 2020a), Also chitosan increase oleanolic acid saponins in *Calendula officinalis* L. (Alsoufi et al. 2019b), and saponin in *Psammosilene tunicoides*, W. C. Wu & C. Y. Wu. (Qiu et al. 2021), ultrasonic waves also increase oleanolic acid in *Calendula offcinalis* L. (Alsoufi et al. 2019a).

To our knowledge, there has been no report on the hairy root induction and production of galegine in the hairy root culture of *G. officinalis*. Therefore, it aimed to investigate the effect of SA, chitosan, and ultrasonic waves on galegine accumulation in hairy root cultures of this plant.

## Material and methods

### Hairy roots induction

**Fig. 1:**
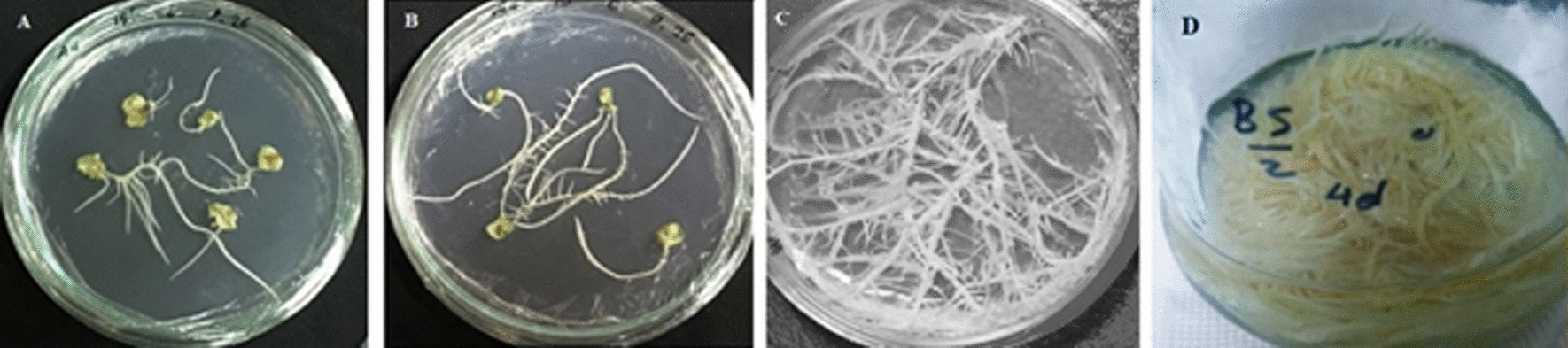
Hairy roots obtained from leaf explants (**a**) and cotyledons (**b**) inoculated for 15 min with the *R. rhizogenes* strain A4, 3-week-old roots obtained from inoculation of cotyledon explants with the strain *R. rhizogenes* A4 (**c**). HR-G3 line of hairy root in liquid 1/2 B5 culture medium (**d**)

Three-week-old sterile seedlings as explants source were used to induce hairy roots. Leaf and cotyledon explants were inoculated with A4, A13, and 15834 strains of *R. rhizogenes* suspension for 10, 15, and 20 min. The explants were then placed in the growth room in the dark on 1/2 B5 solid culture medium, (pH = 5.8), containing 150 µM acetosyringone. 48 h after inoculation, the inoculated explants were washed with sterile water containing 500 mg/L cefotaxime, three times to remove excess bacteria. After removing excess water with sterile filter paper, explants were placed on a solid culture medium containing 300 mg/L cefotaxime and placed in a dark growth room until roots were observed. The induced roots were then transferred to liquid 1/2 B5 culture medium on a shaker at 110 rpm (Fig. 1d). After several subcultures, the roots were transferred to an antibiotic-free medium. The transgenicity of the roots was determined by polymerase chain reaction (PCR).

### Molecular confirmation of transformed hairy roots

Genomic DNA was extracted from hairy root lines using a CTAB-based method (Pirttilä et al. 2001). The polymerase chain reaction was performed to confirm the presence of *rolB* and *rolC* genes and to ensure the absence of *virG* in potential transgenic roots. So, to prepare 20 µL of PCR reactions, 0.5 µL of each forward and reverse primers (Table 1) at a concentration of 10 µmol, 10 µL of PCR master mix (Sinaclon, Tehran), 1 µL of genomic DNA, and 8 µL of nuclease-free water were used. PCR was performed in a thermal cycler according to the following thermal program: 95 °C for 5 min, 35 cycles of 95 °C for 60 s, 59 °C for 60 s, and 72 °C for 90 s, followed by 72 °C for 5 min. The PCR products were observed and examined after electrophoresis on a 1.5% agarose gel under Gel-Doc.

**Table 1:** The sequences of primers used to determination of transgenic roots

Gene name	PCR product length (bp)	Primer sequences
*rolB*	592	5′-CTCACTCCAGCATGGAGCCA-3′ 5′-ATTGTGTGGTGCCGCAAGCTA-3′
*rolC*	473	5′-TGGAGGATGTGACAAGCAGC-3′ 5′-ATGCCTCACCAACTCACCAGG-3′
*virG*	319	5′-AGTTCAATCGTGTACTTTCCT-3′ 5′-CTGATATTCAGTGTCCAGTCT-3′

### Elicitor preparation and treatment

To preparation of elicitors, 0.138 g salicylic acid (Sigma-Aldrich, Münich, Germany) was first dissolved in 100 μL 1 N NaOH and made up to 1 mL with sterile distilled water, then passed through a 0.45-micron nitrocellulose filter and used in hairy root cultures at concentrations of 100, 200 and 300 μM. The stock of 1000 mg/L of water-soluble chitosan (Chitoplant; Sigma-Aldrich, Münich, Germany) was prepared and filtered through a 0.45-micron nitrocellulose filter before use. The filtered chitosan was applied to the hairy root cultures at concentrations of 100, 200, and 400 mg/L. Also for ultrasonic treatment, the hairy roots were placed in the ultrasonic bath (Bandelin, DT 102 H, 35 kHz, 320 W, Belgium) for 1, 2, and 4 min. Subsequently, the elicited hairy roots were placed on a 110 rpm shaker at room temperature in the dark. After 2 and 4 days, the hairy roots were harvested. After harvest, excess water was removed from the roots using Whatman filter paper and the roots were weighed with an analytical balance (AND Weighing FX-5000i Precision Balance 5200 × 0.01 g).

### H_2_O_2_ and MDA contents

The method of Sergiev et al. (1997) was used to measure H_2_O_2_ and MDA contents. For this, 1.5 mL of 1% trichloroacetic acid (pH = 7.0) was added to 100 mg of hairy roots powder and the samples were centrifuged at 12,000 rpm for 15 min at 4 °C. Finally, to measure of H_2_O_2_ content, 0.5 mL aliquot of the supernatant was added to 0.5 mL of 10 mM PBS (pH = 7.0) and 1 mL of 1 M potassium iodide. For the blank, 0.5 mL of extraction solution was used in place of the extract. Absorbance was recorded with a spectrophotometer at 390 nm. A standard curve with known H_2_O_2_ concentrations was used to calculate the amount of H_2_O_2_ content.

The thiobarbituric acid (TBA, Sigma-Aldrich) according to Heath and Packer (1968) method was used to determine malondialdehyde (MDA) production as a measure of lipid peroxidation of hairy roots. For this purpose, 0.5 mL of the extract was added to 1 mL of the reaction solution (containing 20% trichloroacetic acid and 0.5% thiobarbituric acid) and incubated in a boiling water bath (100 °C) for 30 min. The samples were then immediately placed on ice and centrifuged at 10,000 rpm for 15 min at 4 °C. The absorbance of the supernatant was recorded at 532 nm and corrected for nonspecific absorbance at 600 nm using a UV–vis spectrophotometer. The amount of malondialdehyde (MDA) was calculated by using an extinction coefficient of 155 mM^−l^ cm^−l^ (Heath and Packer 1968).

### Determination of the phenol, flavonoid, and galegine contents

Zero point three gram of the hairy roots powder was mixed with 10 mL of HPLC grade methanol (Merck-Germany). The samples were shaken at 110 rpm (25 °C) for 30 min and then sonicated in an ultrasonic bath (Bandelin, DT 102 H, 35 kHz, 320 W, Belgium) for 40 min at room temperature. Samples were concentrated to 700 μL after straining with Whatman filter paper (Class 42) at 50 °C in an oven. The extracts were then passed through a 0.45-micron polyvinylidene fluoride (PVDF) membrane filter and used to determination of the total phenol and flavonoid contents as described before in Khezri et al. (2022). Also, galegine content was determined using the HPLC method modified from Raigond et al. (2018) and described before in Khezri et al. (2022). Briefly, To measure the amount of galegine in control and treated samples, a reversed-phase C18 column (250 mm × 4.6 mm, 5 μm particle size) was used in the YL9100 HPLC system (YoungLin Clarity, South Korea), which consisted of YL9120 UV/Vis detector with Rheodyne 9725i manual sample injector. The determination of galegine was carried out at a column oven temperature of 25 ± 2 °C with a flow rate of 1.0 mL/min in the isocratic system using 0.05 M potassium dihydrogen phosphate (pH = 3.5) and 100% acetonitrile (at a ratio of 70–30) as the mobile phase. UV–vis detector was set at 232 nm. The 20 μL of each sample was injected into the HPLC system. The concentration of galegine in the samples was identified and quantified using the standard quantification method (ESTD) and a standard curve (y = 64.702 x + 422.83; R^2^ = 0.998) was constructed using galegine (AKOS GmbH—AKOS000276789, Germany).

### Statistical analysis

The effects of different concentrations and durations of elicitation on fresh weight and biochemical characteristics were statistically analyzed as a factorial experiment based on a completely randomized design (CRD). All data were obtained from at least three replicates. Means were compared by Duncan’s multiple range test (*p* ≤ 0.01) using SPSS 16.0 software.

## Results

### Hairy roots induction

Hairy roots emergence at wounded sites of inoculated explants was observed in 5–20 days after co-cultivation. Leaf and cotyledon explants of *G. officinalis* were transformed with a wild strain of *R. rhizogenes* A4, ATCC 43057™ (Fig. 1a, b). The results were not satisfactory for strains A13 and 15834. The highest induction rate (58.32%) and the most number of roots (2.61 roots per explant) were obtained from the leaf explants co-cultivated with A4 strain for 15 min.

### PCR amplification

**Fig. 2:**
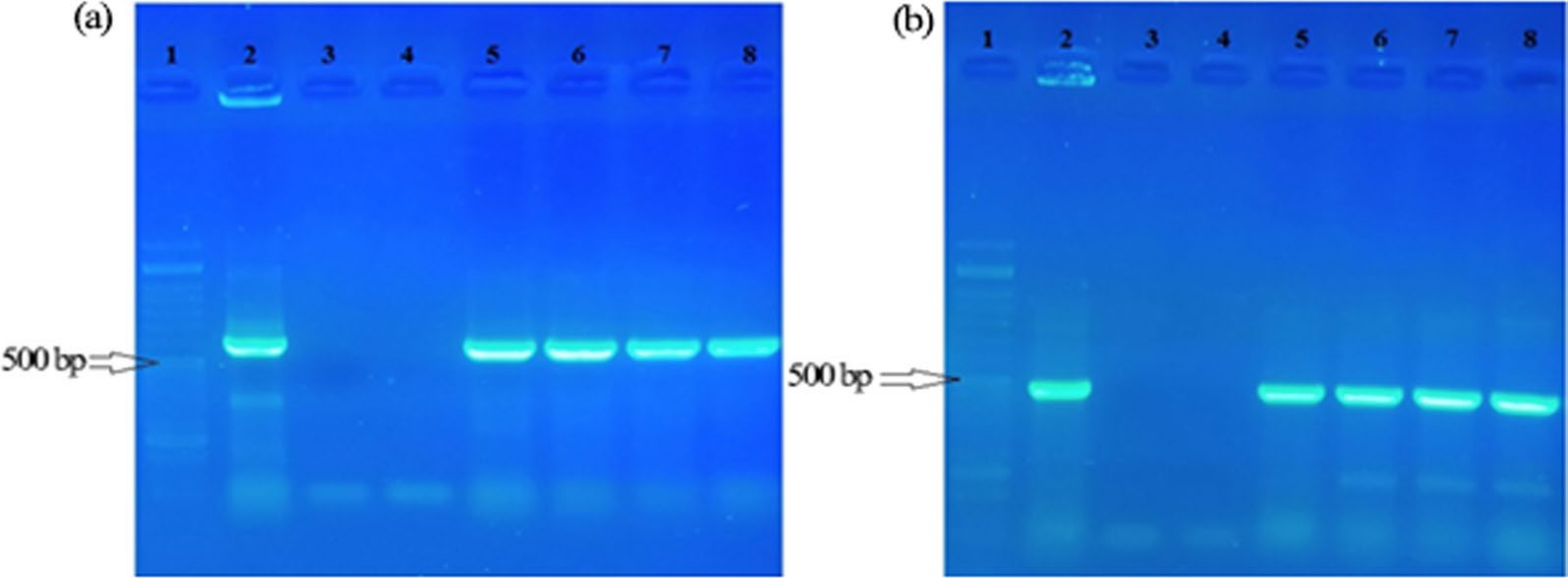
PCR analysis of hairy roots obtained by inoculation with the A4 strain of *R. rhizogenes* using specific primers for the *rolB* gene (**a**) and the *rolC* gene (**b**). Lanes 1, 50 bp DNA ladder (Sinaclone); lane 2, Ri plasmid of *R. rhizogenes* as positive control; lane 3 and 4, DNA-free PCR reaction as negative control; Lanes 5, 6, 7, and 8, Hairy roots from inoculation

**Fig. 3:**
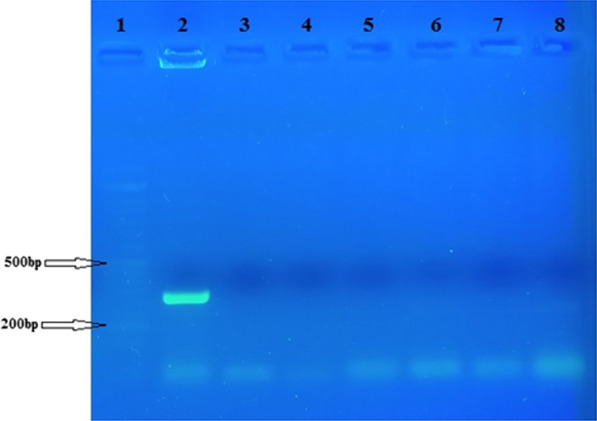
PCR analysis of hairy roots using the *virG*-specific gene primer. Lane 1, 50 bp DNA ladder (Sinaclone); lane 2, Ri plasmid of *R. rhizogenes* as positive control; lane 3 and 4, DNA-free PCR reaction as negative control; lanes 5, 6, 7, and 8, hairy roots from inoculation

The presence of the *rolB* (592 bp Fig. 2a) and *rolC* (473 bp Fig. 2b) genes and the absence of *virG* (319 bp Fig. 3) in the transformed roots of *R. rhizogenes* were confirmed by PCR analysis using specific primers (Table 1). The *R. rhizogenes* plasmid was used as a positive control.

### Determination of best growing hairy root line

**Fig. 4:**
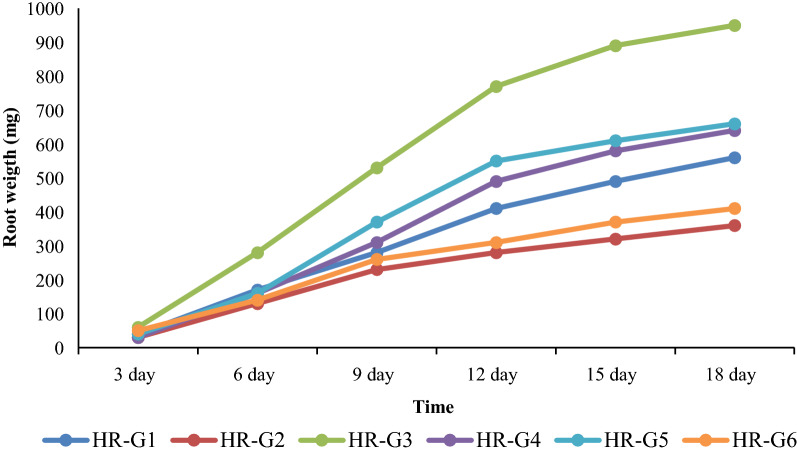
Comparison diagram of growth of six hairy root lines during several subcultures. The growth rate is based on fresh weight changes

To determine the best hairy root lines in terms of growth rate, the high growing lines were weighed every three days and a growth curve was plotted. According to the results, the line HR-G3 obtained from the cotyledon was chosen as the best line (Figs. 1c; 4) and it was subcultured once a week. Then, at the end of the growth log phase (approximately 10th day), chitosan (100, 200, and 400 mg/L), salicylic acid (100, 200, and 300 µM), and ultrasound (1, 2, and 4 min) were applied. The roots were harvested two and four days after elicitation.

### Growth characteristics

**Fig. 5:**
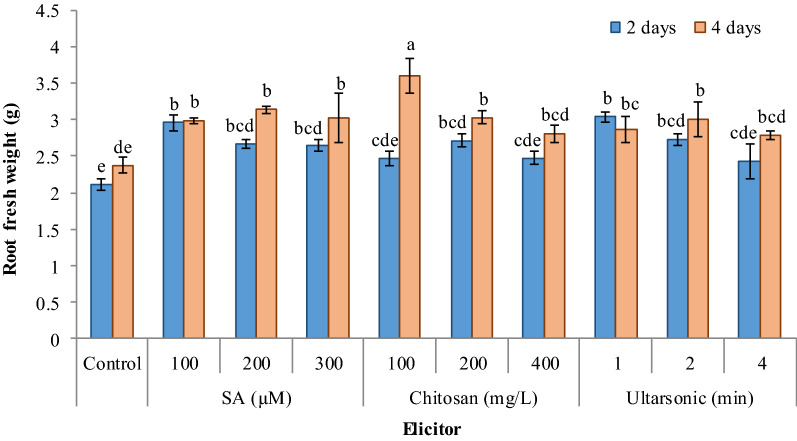
Effect of different elicitors on fresh weight in hairy root culture of *G. officinalis*. Different letters show significant differences (*p* < 0.01) among treatments

The results showed that the fresh weight was increased significantly in most treated hairy roots as compared with untreated roots. Among the elicitors, the application of 100 mg/L chitosan for 4 days had the best positive effect on hairy root weight (Fig. 5). Salicylic acid at all concentrations significantly increased fresh weight as compared with untreated roots. For roots treated with chitosan for 2 days, only 200 mg/L resulted in a significant increase in fresh root weight, but at 4-day treatment increasing chitosan concentration caused in reduction of hairy root weight. As so at the highest concentration (400 mg/L), the difference was not significant as compared with untreated roots. For ultrasonic, at its highest intensity (4 min) there was no significant difference treated hairy root’s fresh weight in comparison with the untreated roots, but the lower intensities for both the 2- and 4-day treatments significantly increased the fresh weight of the roots.

### H_2_O_2_ and MDA contents

**Fig. 6:**
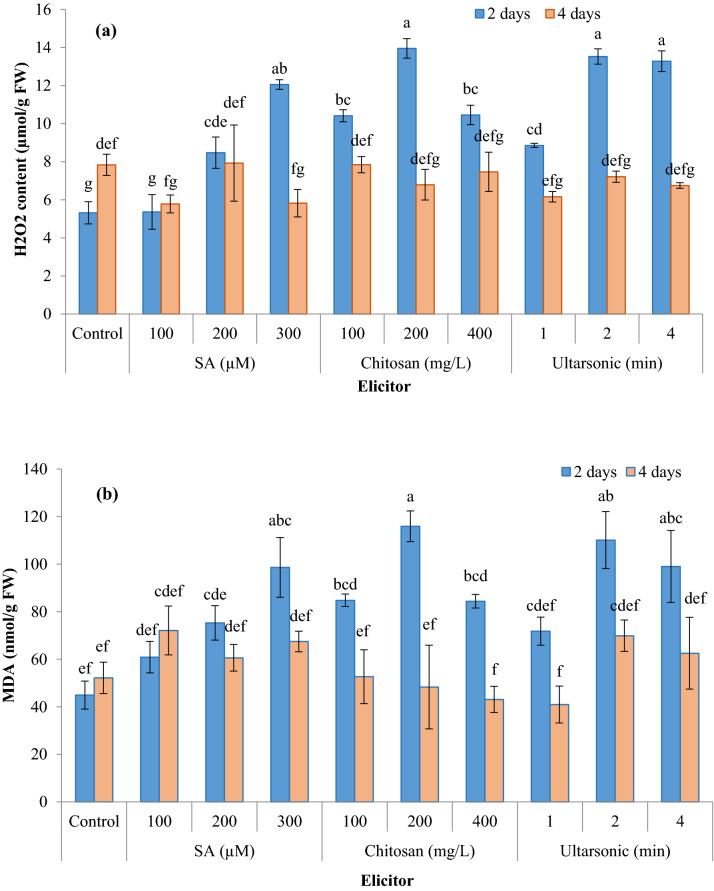
Effect of different elicitors on H_2_O_2_ (**a**), and MDA (**b**) in hairy root culture of *G. officinalis*. Different letters show significant differences (*p* < 0.01) among treatments

To investigate the ROS burst and scavenging in response to the elicitor treatments, H_2_O_2_ and MDA content were determined in hairy roots treated with chitosan, SA, and ultrasound. The amount of MDA and H_2_O_2_ increased significantly in hairy roots treated for two days with all three elicitors, except for 100 μM SA, compared with untreated roots. However, their levels in hairy roots harvested after four days showed no significant change in any of the treatments compared to untreated roots. The highest MDA and H_2_O_2_ contents were obtained when hairy roots were treated with 200 mg/L chitosan for 2 days without significant differences with 300 μM SA, and 2 and 4 min treatment with ultrasound (Fig. 6).

### Total phenol and flavonoids

**Fig. 7:**
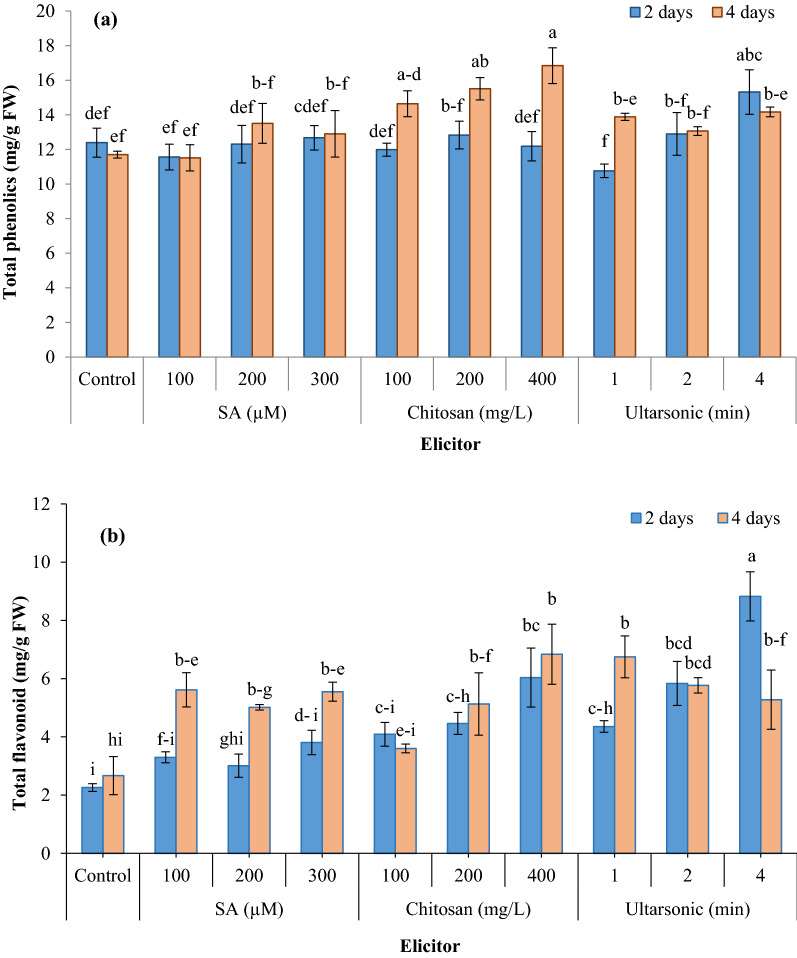
Effect of different elicitors on total phenol (**a**), and total flavonoid (**b**) in hairy roots of *G. officinalis*. Different letters show significant differences (*p* < 0.01) among treatments

The results showed that the at 2-day treatment only the hairy roots treated with ultrasound for 4 min had a significantly higher phenolic content (15.32 mg/g FW) than the control, and the other treatments had no significant effect. Similarly, the 4-day application of all salicylic acid concentrations and also ultrasonic waves for 1, 2, and 4 min, did not result in a significant increase in total phenolic content as compared with untreated roots (Fig. 7a). 2-day application of chitosan did not lead to a significant increase in total phenolic content in treated roots (Fig. 7a). However, the 4-day application of chitosan at all concentrations (100, 200, and 400 mg/L) resulted in a significant increase in the total phenolic content in the treated hairy roots as compared with the untreated ones.

The application of salicylic acid for 2 days did not significantly increase total flavonoid content in hairy roots compared with untreated roots. However, its application at all concentrations studied for 4 days resulted in a significant increase in total flavonoid content of hairy roots in comparison with the untreated roots (Fig. 7b). On the other hand, 100 mg/L of chitosan did not cause a significant increase in total flavonoid content in hairy roots treated both for 2 or 4 days, while at 200 and 400 mg/L it caused a significant increase in total flavonoid content at both 2 and 4 days. Hairy roots treated with ultrasound for 1, 2, and 4 min had significantly higher flavonoid contents than untreated ones after both 2 and 4 days. It was observed that an increase in ultrasound duration resulted in an increase in flavonoid content at the 2-day treatment, but at the 4-day treatment, an increase in ultrasound duration result in a decrease in total flavonoids, however this decrease did not significant. As so, the highest flavonoid content was obtained in hairy roots that treated with ultrasound for 4 min and harvested two days later (8.82 mg/g FW).

### Galegine content

**Fig. 8:**
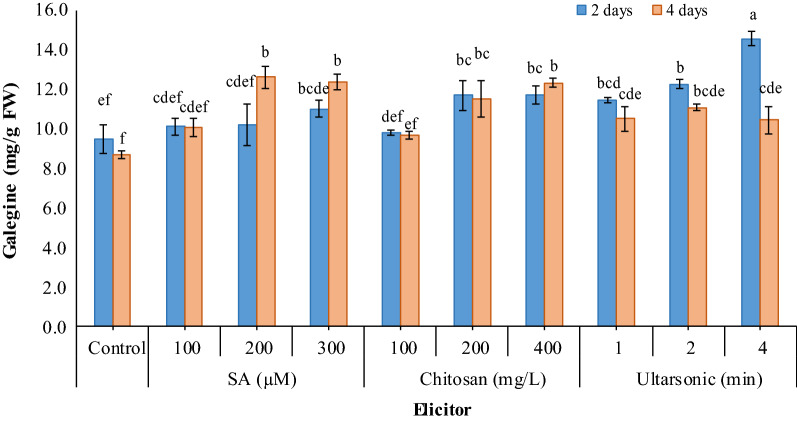
Effect of different elicitors on galegine content in hairy root culture of *G. officinalis.* Different letters show significant differences (*p* < 0.01) among treatments

**Fig. 9:**
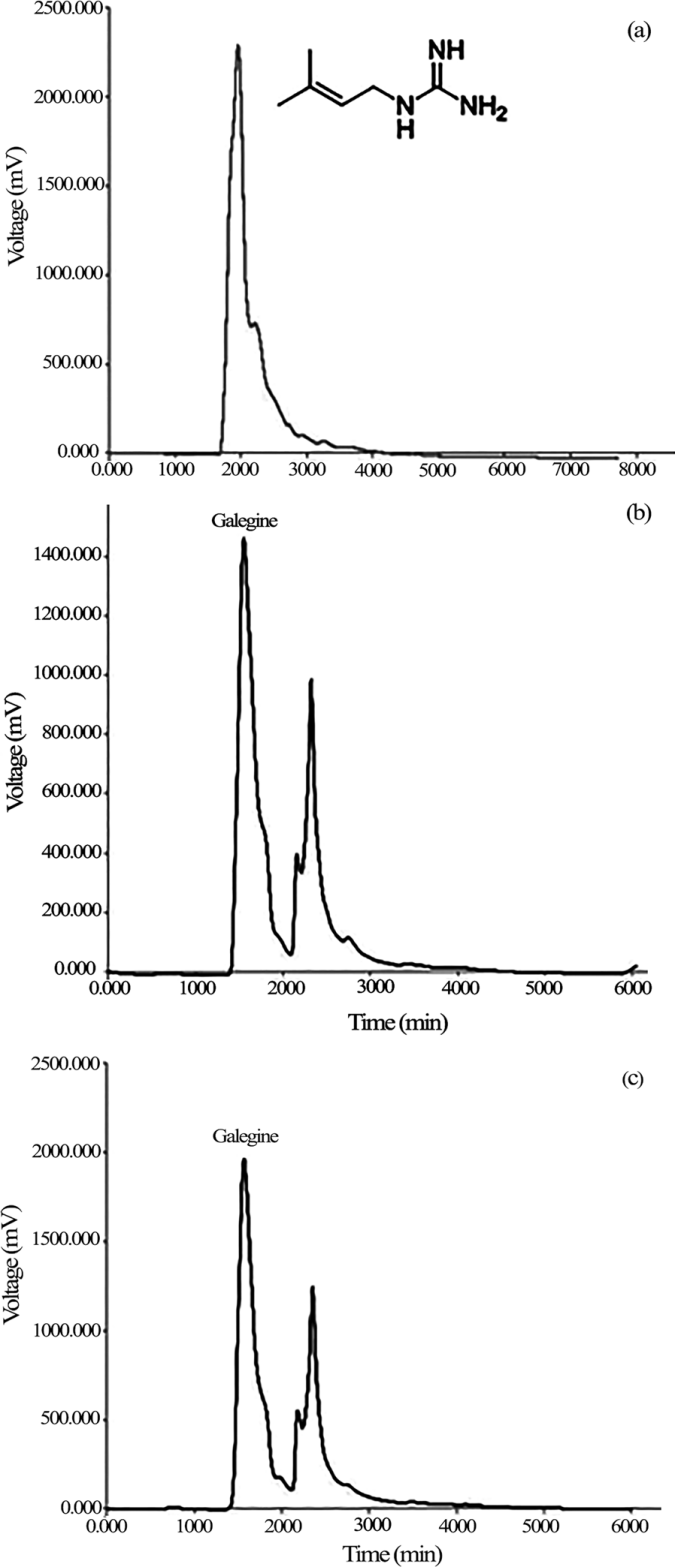
HPLC chromatogram showing galegine standard (**a**) galegine content in untreated hairy roots (**b**), and in roots treated with ultrasonic waves for 4 min in hairy root culture of *G. officinalis* (**c**)

HPLC analysis revealed a very significant (*p* < 0.01) difference between treated and untreated hairy roots in galegine content (Fig. 8). The presence of galegine was confirmed by retention time (approximately 1.4) using the galegine standard (Fig. 9). Treatment of hairy roots for 2 days with any of the salicylic acid concentrations did not significantly increase the galegine content. However, treatment of the roots with 200 and 300 µM of the elicitor for 4 days significantly increased the galegine content (12.58 and 12.38 mg/g FW, respectively). Also, the galegine content of hairy roots in treatment with 200 and 400 mg/L chitosan for both two and four days was increased, but no significant increase was observed in hairy roots treated with 100 mg/L chitosan. In comparison with the control, hairy roots treated with ultrasound for 1, 2, and 4 min had a significantly higher content of galegine harvested both 2 and 4 days after. Although the galegine content in hairy roots harvested four days after treatment decreased, the amount was still higher than untreated roots. Overall, the highest amount of galegine was obtained in hairy roots treated with ultrasound for 4 min and harvested 2 days after elicitation (14.55 mg/g FW, Fig. 8)**.**

## Discussion

Elicitors are chemical or biological factors derived from various sources that are capable of inducing physiological changes in living organisms (Zhao et al. 2005). Elicitation is a potential tool to overcome various difficulties associated with the production of most commercially important bioactive secondary metabolites from wild and cultivated plants, undifferentiated or differentiated tissues (Halder et al. 2019). The application of methyl jasmonate, jasmonic acid, salicylic acid, abscisic acid, and other signaling molecules can inhibit growth and thus affect the amount of biomass and secondary metabolites (Halder et al. 2019; Srivastava and Srivastava 2014; Wang and Wu 2013). According to the literature, the response of hairy roots to elicitation is highly variable among different plants and elicitors. Thus, we observed that in most treatments used, fresh weight of hairy root culture of *G. officinalis* was increased by elicitation with SA, chitosan, and US. While SA positively affected the growth of hairy root culture of *Agastache foeniculum* (Pursh) Kuntze (Nourozi et al. 2014) and *Tripterygium wilfordii* Hook F (TwHF) (Halder et al. 2019; Zhu et al. 2014) compared to the control, chitosan treatment negatively affected the growth rate of hairy roots and both fresh and dry mass of in vitro hairy roots of *Calendula officinalis* L. (Alsoufi et al. 2019b). In contrast, in some species, including orchids (Ferri and Tassoni 2011) and *Agastache foeniculum* (Nourozi et al. 2014) chitosan acts as a growth promoter.

In the present study, the application of elicitors in the culture medium of hairy roots resulted in a concentration-dependent increase in the content of H_2_O_2_ and MDA, total phenol and flavonoid, and galegine compared with untreated roots. As in most cases, the application of higher concentrations of salicylic acid for two days in hairy root culture significantly increased the content of H_2_O_2_ and MDA, and application for four days did not significantly change the content of these factors. The content of galegine and flavonoids in roots treated with salicylic acid for 4 days was higher, and this increase was significant compared to untreated roots. The amount of total phenol in most of treated hairy roots did not change significantly compared to untreated roots.

Some studies reported that induction of oxidative stress was observed in plants exposed to methyl jasmonate (MJ) and SA (Di-Qiu et al. 1999; Ho et al. 2020; Mir et al. 2019). Also, treatment with SA increased photosynthesis and activated antioxidant enzyme activity in *Cannabis sativa* L. (Pilaisangsuree et al. 2020; Shi et al. 2009). Exogenous SA also can induce the activity of antioxidant enzymes and the expression of related genes, as well as the formation of pathogen-related proteins (PR) in plants (Mejía-Teniente et al. 2013). Salicylic acid (SA) causes systemic acquired resistance (SAR) in plants. SAR is a physiological state induced by specific environmental stimuli, thereby increasing the plant's defense capacity against biological stress (van Loon 2016). At the molecular level, SAR is generated in local and systemic tissues by increasing the expression of a large number of pathogenic gene families. These pathogenesis related (PR) genes encode a heterogeneous group of low molecular weight proteins that are induced by chemical stimuli (Tripathi et al. 2019).

The inefficiency of SA in promoting secondary metabolites production in the roots of *Valeriana amurensis* (Cui et al. 2012), *Valeriana jatamansi* Smir. ex Kom. (Shuang and Hong 2020), *Gentiana dinarica* Jones (Krstić-Milošević et al. 2017) has also been reported. However, several studies have demonstrated the efficacy of SA in enhancing the target metabolites in plant cells or organ cultures, such as *Isatis tinctoria* L. (Gai et al. 2019), *Salvia przewalskii* Maxim (Li et al. 2020a), and *Papaver armeniacum* L. (Naeini et al. 2021)*.*

According to the results, all concentrations of chitosan applied for 2 days caused a significant increase in H_2_O_2_ and MDA, but their application for four days did not cause a significant change in the amount of these parameters. Conversely, the application of all concentrations of chitosan for four days resulted in a significant increase in total phenols, but the application for two days did not significantly change the amount. The concentrations of 200 and 400 mg/L of chitosan in the 2- and 4-day treatments increased the total flavonoid content and galegine. However, the concentration of 100 mg/L did not significantly change the amount of these parameters. Chitin and chitosan are the structural components of the cell wall of fungi. They are N-acetylglucosamine polymers, mainly glucosamine, and they are among the most abundant polymers on earth (Hadwiger 2013). This material is non-toxic and environmentally friendly. The mechanism of action of chitosan in plants is not yet fully understood. However, many reports are showing that chitosan has triggered several defense responses in plants (Hidangmayum et al., 2019). Biochemical and molecular changes observed in chitosan-treated plants include activation of ROS inhibitor system, membrane lipid peroxidation (Li et al. 2020b), hydrogen peroxide accumulation (Lin et al. 2005), increase in cytosolic Ca^2+^ (Zuppini et al. 2004), hypersensitivity reaction (Hadwiger and Beckman 1980), activation of MAP-kinases (Petutschnig et al. 2010), an increase of mRNA of genes related to PR pathogenesis (Hadwiger 2013; Li et al. 2020b), prevention of excessive transpiration and increase of stomatal conductance, improvement of root and overall plant growth, production of phytoalexins (Hidangmayum et al. 2019), and induction of chitinase and glucanase enzymes involved in pathogen resistance (Hidangmayum et al. 2019; Sathiyabama et al. 2014).

The induction of plant defenses after elicitation with chitosan is usually associated with physiological activities, mainly as antioxidants (e.g., polyphenols) (Ferri and Tassoni 2011). Chitosan seems to effectively increase the content of a variety of polyphenols, flavonoids (i.e., anthocyanins), and antioxidants, which are very important for plants (Ferri and Tassoni 2011). In a similar study it was observed that the addition of chitosan to the culture medium of *Plumbago indica* L. hairy roots resulted in the greatest increase in plumbagin production at a concentration of 200 mg/L (Gangopadhyay et al. 2011;). Moreover, (Qiu et al. 2021) reported that in the hairy root culture of *Psammosilene tunicoides* W. C. Wu & C. Y. Wu, the highest total saponin accumulation occurred in the roots induced by 200 mg/L chitosan for 9 days (Qiu et al. 2021). Alsoufi et al. (2019b) also reported that chitosan in hairy root cultures of *Calendula officinalis* L. increased the accumulation and secretion of oleanolic acid saponins up to three-fold (Alsoufi et al. 2019b).

According to the studies, the application of chitosan particles of different sizes in wheat seedlings under salt stress significantly reduced MDA concentration, increased chlorophyll and proline content, enhanced photosynthesis, activated antioxidant enzyme activity such as SOD, POD and CAT (Hidangmayum et al. 2019). Also, chitosan at different concentrations increases H_2_O_2_ and MDA content in scab culture of *Digitalis lanata* Ehrh. (Pérez-Alonso et al. 2012). Exogenous application of chitosan dramatically triggered the content of reactive oxygen species (ROS) scavenging enzyme activities in the hairy roots of *Psammosilene tunicoides* (Qiu et al. 2021).

Ultrasound-treated roots harvested four days after the treatment caused no significant change in the content of total phenolic, H_2_O_2_, and MDA, whereas all ultrasound treatments resulted in a very significant increase in the contents of total flavonoids and galegine. Ultrasound (US) is a special physical elicitor with various biological effects that can generally damage biological materials at high density and stimulate biological activities such as enzymatic and microbial biological transformations and cell biosynthesis at low density (Erte et al. 2021). Ultrasonic waves can significantly increase the synthesis of secondary metabolites, depending on the plant species (Dörnenburg and Knorr 1995; Taherkhani et al. 2019). So, US induces cell membrane permeability (Knorr 2003), and produces a stress response similar to that induced by wound stress. In this way, the application of US releases ATP from the damaged cells, and distributes it through the intercellular spaces and binds it to the ATP receptors in the plasma membrane of the damaged cells. The binding of ATP to its receptors generates secondary signaling molecules. These secondary signals are transmitted through the cytosol and initiate the signal transduction network that leads to the activation of transcription factors that cause nutrient biosynthesis (Jacobo-Velázquez et al. 2017).

Wu and Lin (2002) reported that ultrasound treatment caused cross-membrane ion flux, production of reactive oxygen species (ROS), and a rapid increase in phenylalanine ammonia lyase activity (PAL), followed by increased production of polyphenols (PP), and phenolic compounds in the cell suspension of *Panax ginseng* C.A. Meyer. Induced production of phenolic secondary metabolites was also reported in the cell cultures of *Morinda citrigolia* L. (Komaraiah et al. 2005), hazelnut (Rezaei et al. 2011), and *Vitis vinifera* L. (Santamaria et al. 2012). In some studies, treatment with high-intensity ultrasound has been reported to stimulate the accumulation of secondary metabolites and increase antioxidant activity in agricultural products such as strawberries (Gani et al. 2016), peanuts (Sales and Resurreccion 2010) and rice sprouts (Ding et al. 2018a) and wheat (Ding et al. 2018b).

At all, it was concluded that to increase the content of secondary metabolites in the hairy root culture of *G. officinalis*, the application of elicitors is an effective strategy. Our results showed that among the elicitors, the application of ultrasonic waves for 4 min was more effective to increase the content of the flavonoids and galegine.

## Data Availability

All the data generated or analyzed during this study are included in this published article (and its additional information files).
